# Prioritizing industries for occupational injury prevention and research in the Services Sector in Washington State, 2002–2010

**DOI:** 10.1186/s12995-014-0037-2

**Published:** 2014-11-09

**Authors:** Naomi J Anderson, David K Bonauto, Darrin Adams

**Affiliations:** Washington State Department of Labor & Industries, Safety & Health Assessment & Research for Prevention (SHARP) Program, PO Box 44330, Olympia, WA 98504-4330 USA

**Keywords:** Surveillance, NORA sector, Workers’ compensation, Traumatic injury, Work related musculoskeletal disorder, Falls, Motor vehicle, Occupational health, Safety

## Abstract

**Background:**

The Services Sector, as defined by the National Occupational Research Agenda (NORA), is comprised of a diverse industry mix and its workers face a variety of occupational exposures and hazards. The objective of this study was to identify high-risk industry groups within the Services Sector for prevention targeting.

**Methods:**

Compensable Washington State workers’ compensation claims from the Services Sector from 2002 through 2010 were analyzed. A “prevention index” (PI), the average of the rank orders of claim count and claim incidence rate, was used to rank 87 Services Sector industry groups by seven injury types: Work- Related Musculoskeletal Disorders (WMSDs), Fall to Lower Level, Fall on Same Level, Struck By/Against, Caught In/Under/Between, Motor Vehicle, and Overexertion. In the PI rankings, industry groups with high injury burdens appear higher ranked than industry groups with low counts or low rates of injury, indicating a need for prioritizing injury prevention efforts in these groups.

**Results:**

In the Services Sector, these 7 injury types account for 84% of compensable claims in WA. The industry groups highest ranked by PI across the injury types included: Services to Buildings and Dwellings; Executive, Legislative, and Other General Government Support; and Waste Collection. WMSDs had the highest compensable claims rates.

**Conclusions:**

Services is a large sector of the economy, and the substantial number, rate, and cost of occupational injuries within this sector should be addressed. Several Services Sector industry groups are at high risk for a variety of occupational injuries. Using a PI to rank industry groups based on their injury risk provides information with which to guide prevention efforts.

## Background

Occupational injuries and illnesses are common, costly, and a burden to workers and employers. Resources for prevention are limited, and there is a need for information to better focus research and prevention activities to maximize their impact. Other than the Bureau of Labor Statistics Survey of Occupational Injuries and Illnesses [1], there is relatively little published surveillance data comparing occupational injury and illness rates across industries [2-5]. In order to effectively prevent occupational injuries, employers, policymakers, healthcare providers, and researchers must know which injuries are occurring, where, and to what extent (magnitude, associated costs and time-loss). Analyzing injuries by industry can identify workers at high-risk for occupational injuries, and industry groups with the greatest need for prevention activities, safety and health programs, and further research.

The second-decade National Occupational Research Agenda (NORA) [6] is a partnership program between the National Institute for Occupational Safety and Health (NIOSH) [7] and businesses, universities, labor and other stakeholders, to promote and improve occupational health and safety research and workplace practices. The North American Industry Classification System (NAICS) classifies establishments into industries based on their business activities (and similarity in processes used to produce goods or services) [8]. NORA aggregates the 20 NAICS 2-digit Industry sectors into 10 Sector groups [9].

The Services Sector, as defined by NORA, includes diverse industries that face a range of exposures and hazards (e.g. offices, banks, educational establishments, auto repair shops, science and technical work, restaurants, fast food, hotels and motels, waste collection and the performing arts) and vary in establishment size and work organization. The Services Sector encompassed approximately 65 million workers in 2011, or more than 50% of the total U.S. workforce [10]. The scope and diversity of this Sector makes targeting occupational safety, prevention and research efforts difficult. Workers in the Services Sector may also face a higher burden of occupational injury and illness [5] and of occupational fatalities [11].

Previous efforts in Washington State (WA) used workers’ compensation (WC) data to prioritize WA industries for injury prevention by ‘prevention index’ (PI) rankings for common costly occupational injury types and identified several NAICS industry groups that ranked highly on the prevention index [2,12]. The prevention index is calculated as the average of the industry’s rank order of WC compensable claim count and compensable claims rate. In other words, the PI ranks industries by equally weighting how common injuries are and how high a worker’s risk for injury is. Industry groups that are ranked higher by PI are those with a greater burden or risk of injuries. PI rankings indicate which industry groups should be prioritized in targeting research and prevention.

In one study, Anderson, Bonauto, and Adams [12] used PI methodology to rank all industry groups in WA (limited to State Fund claims) between 2002–2010. Of the 262 industry groups that met the inclusion criteria, 5 of the top 25 industry groups represented were from the Services Sector (Table 1). Two Services Sector industry groups, Services to Buildings and Dwellings (NAICS 5617) and Waste Collection (NAICS 5621) were in the top 5 by claim rate or count overall. These findings identified the Services Sector as a candidate for more in-depth characterization.

**Table 1:** **Top 25 NAICS industry groups by prevention index for WA State fund (all sectors, all injury types), 2002-2010**

**NORA sector**	**4-digit NAICS (Industry group)**	**NAICS industry group description**	**FTE**	**# Compensable claims (COUNT)**	**Compensable claim rate/10,000 FTE (RATE)**	**Median compensable cost**	**Median days TL**	**Severity TL (days/10,000 FTE)**	**Rate rank (All SF)**	**Count rank (All SF)**	**Overall SF PI rank**
C	2381	Foundation, structure, and building exterior contractors	137,685	9,312	676.3	$13,196	68	161,159	2	3	1
C	2361	Residential building construction	186,292	9,792	525.6	$11,280	57	129,804	10	2	2
C	2383	Building finishing contractors	160,942	8,281	514.5	$13,697	71	136,950	11	4	3
U	4841	General freight trucking	89,627	4,985	556.2	$10,779	55	137,877	8	11	4
C	2382	Building equipment contractors	276,061	12,495	452.6	$17,782	77	105,487	18	1	4
H	6232	Residential mental retardation, mental health and substance abuse facilities	48,764	3,251	666.7	$6,354	33	115,168	3	19	6
A	1133	Logging	35,322	2,642	748.0	$14,347	66	204,306	1	26	7
C	2389	Other specialty trade contractors	123,197	5,456	442.9	$13,214	62	111,986	21	9	8
U	4842	Specialized freight trucking	46,765	2,685	574.2	$9,130	49	116,432	7	25	9
**S**	**5617**	**Services to buildings and dwellings**	**192,258**	**7,860**	**408.8**	**$7,489**	**43**	**82,849**	**30**	**5**	**10**
H	6231	Nursing care facilities	122,719	4,437	361.6	$6,649	33	63,675	41	12	11
**S**	**5621**	**Waste collection**	**21,310**	**1,316**	**617.6**	**$7,665**	**34**	**90,504**	**5**	**53**	**12**
M	3219	Other wood product manufacturing	48,441	2,032	419.5	$8,869	30	74,729	26	32	12
C	2373	Highway, street, and bridge construction	45,004	1,880	417.7	$30,101	108	109,069	27	35	14
H	6243	Vocational rehabilitation services	55,612	2,179	391.8	$5,206	28	60,168	35	30	15
C	2371	Utility system construction	57,242	2,147	375.1	$19,443	78	96,020	38	31	16
C	2362	Nonresidential building construction	108,886	3,580	328.8	$25,025	94	72,752	54	16	17
**S**	**9221**	**Justice, public order, and safety activities**	**137,032**	**4,149**	**302.8**	**$9,360**	**30**	**37,377**	**65**	**13**	**18**
M	3211	Sawmills and wood preservation	26,888	1,131	420.6	$9,902	34	83,822	24	58	19
**S**	**8111**	**Automotive repair and maintenance**	**132,964**	**3,730**	**280.5**	**$10,053**	**45**	**65,058**	**73**	**15**	**20**
T	4244	Grocery and related product merchant wholesalers	194,917	5,187	266.1	$9,441	43	48,304	81	10	21
M	3323	Architectural and structural metals manufacturing	45,395	1,572	346.3	$10,914	41	57,173	49	44	22
H	6233	Community care facilities for the elderly	96,742	2,740	283.2	$7,686	39	56,851	71	24	23
U	2213	Water, sewage and other systems	109,958	3,060	278.3	$9,755	26	30,818	74	22	24
**S**	**5613**	**Employment services**	**245,383**	**6,118**	**249.3**	**$5,687**	**42**	**44,743**	**92**	**8**	**25**

NORA Sector key: *A* = Agriculture, Forestry & Fishing; *C* = Construction; *H* = Healthcare & Social Assistance; *M* = Manufacturing; *S* = Services; *U* = Transportation, Warehousing & Utilities; *T* = Wholesale & Retail Trade. Services Sector industry groups (bold).

There were 262 NAICS Industry Groups ranked in the PI for All Injury Types (State Fund only).

FTE = (hours/2000); Severity TL = (TL days/10,000 FTE).

This study uses the established PI methodology to rank industry groups within the Services Sector by injury type for compensable WC claims between 2002 and 2010. The objective of this study was to identify and prioritize high-risk industry groups in the Services Sector by seven common, high-cost injury types for research and prevention efforts. Looking at PIs for each injury type allows for further tailoring of prevention efforts, as some industry groups may be at high-risk for specific injury types and not others. Industry groups that rank highly by PI across several injury types should be a top priority for those engaged in occupational health and safety.

## Materials and methods

### Washington’s workers’ compensation system

In Washington State, non-federal employers are required to obtain workers’ compensation insurance through the Department of Labor and Industries’ (L&I) industrial insurance system, unless they qualify to self-insure, or are covered by an alternative WC system (e.g. Longshore and Harbor Workers’ Compensation Program). L&I administers the State Fund (SF), an industrial insurance program that provides coverage for approximately two-thirds of the 3.3 million workers in WA. The SF generally does not mandate WC insurance for self-employed workers, though elective coverage is available. Outside of the SF, there are approximately 450 self-insured (SI) entities (individual companies or groups of companies) that are not included in the State Fund insurance pool who employ nearly one-third of the workforce. Data from both SF and SI programs are collected and maintained in centralized databases at L&I.

### Claim coding & data

In WA, a physician and worker initiate a WC claim by filing a Report of Industrial Injury or Occupational Disease (RIIOD) form, which includes the workers’ demographic information, employment and wage information, and a brief description of the incident. The physician provides a medical diagnosis (with ICD-9) code, subjective and objective information regarding the diagnosis, and a diagnostic and treatment plan.

Workers’ compensation administrative data include: codes characterizing the injury or illness; costs associated with disability payments, wage replacement, and pensions; billing information for health care providers, procedures, and treatment; and physician diagnosis codes. Information on SI claims is often incomplete; therefore cost data is only reported for SF claims in this analysis.

Claim costs for closed claims reflect actual paid costs. For claims that are not closed, costs reflect actual totals paid to date plus actuarial estimates for future costs associated with the claim. Indirect costs (to the employer and worker, e.g. lost or reduced productivity, employee turnover, and morale) and the administrative costs of managing claims are not included in the claim costs.

All WA WC SF claims are coded for nature, part of body affected, source and secondary source, and event or exposure leading to injury or illness according to the Occupational Injury and Illness Classification System (OIICS) [13] from the information on the RIIOD form. OIICS codes are only assigned at the beginning of a claim, and represent a description of the injury or illness at that point in time.

Each employer is assigned a NAICS 2002 code [14] identifying the firm’s industry. NAICS groups ‘economic activity’ into 20 *sectors* (two digit code), 100 *subsectors* (three digit code) and 317 NAICS *industry groups* (four digit code) [14]. The Services Sector, as defined by NORA, is comprised of industries within 11 NAICS Sectors: Information (NAICS 51); Finance and Insurance (NAICS 52); Real Estate, Rental, Leasing (NAICS 53); Professional, Scientific, and Technical (NAICS 54); Management of Companies and Entities (NAICS 55); Administrative Support and Waste Management (NAICS 56); Education (NAICS 61); Arts, Entertainment and Recreations (NAICS 71); Accommodations and Food Services (NAICS 72); Other Services (NAICS 81); and Public Administration (NAICS 92).

In WA, the Services Sector accounted for 48.5% of the SF workforce between 2002 and 2010 [12]. The 2010 BLS Geographic Profile of Employment & Unemployment for Washington State provides percentages of employment by certain industry groups; an estimated 62.2% of the WA workforce in 2010 was employed in the Services Sector (based on aggregate estimates from the industry groups that comprise the Services Sector as defined by NORA) [15].

### Data ascertainment

We identified all WC claims within the Services Sector with dates of injury or illness from January 1, 2002 through December 31, 2010. Claims were extracted on December 19, 2012. Data extracted for each claim included claim identification number, claim status (medical only; compensable), OIICS codes for nature, part of body, source, and event or exposure of injury or illness, costs associated with the claim and time loss information. In WA, WC premiums are based on hours of exposure. Employers report hours on a quarterly basis for premium payment. Hours by NAICS industry group were obtained by WC account aggregated over the nine year study period.

A claim is considered a ‘compensable’ claim if it is categorized by the WC system as a ‘compensable’, ‘kept on salary’, ‘total permanent disability’, ‘fatal’ or ‘loss of earning power’ claim. A claim qualifies as ‘compensable’ if it involves payment for time lost from work. Wage replacement payments for time loss commence on the first missed workday following a three day waiting period which does not include the day of injury.

### Injury type

Using OIICS codes, injuries were described by event code (alone or in combination with OIICS nature or body part affected codes and/or ICD-9 codes) and grouped into seven aggregated injury types, which have been described previously [12]. When referring to these aggregated injury type groups in this report, the term “injury type” is used. Claims were analyzed by 7 common injury types that were previously identified as accounting for the majority of compensable claims across all sectors and having significant costs. The injury types were: 1) Work- Related Musculoskeletal Disorders (WMSDs), 2) Fall to Lower Level, 3) Fall on Same Level, 4) Struck By/Against, 5). Caught In/Under/Between, 6) Motor Vehicle, and 7) Overexertion.

Certain injury types were excluded because they each comprised less than 2% of compensable claims (Exposure to Loud Noises; Extreme Temperatures; Bodily Reaction; Abraded; Electrical; Explosion; and Violence). Claims assigned an injury type of ‘Other’ tend to be poorly defined as ‘unclassified/insufficient data’, or ‘accident type not elsewhere classified’, and were also excluded from analysis. SI claims were more frequently coded as ‘Other’. The term “All Injury Types” refers to all injuries, outside of categorization by injury types.

### Data analysis

Descriptive analyses were conducted on compensable claims. A full time equivalent employee (FTE) was defined as working 2,000 hours per year (40 hours per week for 50 weeks per year). Claim rates are expressed as cases per 10,000 FTE. In some instances, a rate of time loss days (TL) per 10,000 FTE was included as a severity measure.

We utilized a prevention index (PI) to rank industries within the Services Sector for prevention and intervention purposes, by 7 high-cost, common occupational injury types. The PI evaluates the frequency and prevalence of claims within an industry group: claim count - how common are the injuries, and claim incidence rate - how high is the worker risk within that industry. The PI is the average of the rank orders of the claim count and claim incidence rate:

$$ \mathrm{PI}=\frac{\mathrm{Frequency}\ \mathrm{Rank}+\mathrm{Incidence}\ \mathrm{Rank}}{2} $$

In case of tie, rate rank was used as the tiebreaker. Industry groups that have high counts and rates (higher injury burden) appear higher ranked in the resultant tables than industry groups with lower counts and rates (lower injury burden).

For determination of the PI for each injury type and to avoid unstable estimates, NAICS industry groups were limited to those who had reported hours in 6 or more years of the study period, with ≥45 compensable WC claims over the period of the study and ≥100 FTE per year during the study period, from 2002 to 2010. There were 87 NAICS Service Sector Industry Groups that met the criteria for inclusion for ‘All Injury Types.’ The top 25 industry groups are presented for ‘All Injury Types’, and the Top 15 are presented for the remaining injury types.

## Results

### Overall description (data not shown)

Between 2002–2010 there were 410,006 compensable claims in WA WC data. Of these 410,006 compensable claims, 267,581 were in the State Fund. Compensable claims in the WA SF between 2002–2010 amounted to $9,923,849,314 dollars in direct WC costs.

Seven common, high cost injury types were analyzed – WMSD, Fall to Lower Level, Fall on Same Level, Struck By/Against, Caught In/Under/Between, Motor Vehicle and Overexertion. These 7 injury types account for 86% of all (SF) compensable claims and 89% of all compensable claim costs (SF only). In the Services Sector, these 7 injury types account for 84% of (combined SF and SI) compensable claims in WA.

In the 87 industry groups that met the ranking criteria in the Services Sector, there were 132,673 compensable claims during the study period. There were 85,183 State Fund compensable claims accounting for 15,937,396 days of time loss (TL) with a median cost of $7,753 (mean cost $35,259). There were 47,490 self-insured compensable claims during the study period.

### All injury types

Within the study period, there were 87 industry groups (4-digit NAICS) within the Services Sector that met the inclusion criteria for ranking for All Injury Types. The top 5 industry groups ranked for All Injury Types (Table 2) were Executive, Legislative, and Other General Government Support (NAICS 9211); Services to Buildings and Dwellings (NAICS 5617); Waste Collection (NAICS 5621); Justice, Public Order, and Safety Activities (NAICS 9221); and Employment Services (NAICS 5613). These were followed closely (tie for 5^th^ rank) by Automotive Repair and Maintenance (NAICS 8111). These industry groups were consistently ranked in the top 12 by PI for all 7 injury types.

**Table 2:** **Top 15 industry groups by prevention index for all injury types within the services sector in WA (SF + SI), 2002 - 2010**

**4-digit NAICS (industry group) & industry group description**		**State fund only**			**All (State fund + Self insured)**					
		**Median comp claim cost (SF Only)**	**Median days TL (SF only)**	**Severity TL (days/10,000 FTE)**	**FTE (SF + SI)**	**# Compensable claims (COUNT) (SF + SI)**	**Compensable claim rate/10,000 FTE (RATE) (SF + SI)**	**Count rank (SF + SI)**	**Rate rank (SF + SI)**	**PI rank**
9211	Executive, legislative, and other general government support	$9,049.00	36	28533.8	655654	24136	368.1	1	4	1
5617	Services to buildings and dwellings	$6,760.00	43	84570	215906	8696	402.8	3	3	2
5621	Waste collection	$6,576.00	34	92058.3	38768	2252	580.9	10	1	3
9221	Justice, public order, and safety activities	$8,618.00	29	38268	137032	4155	303.2	7	8	4
5613	Employment services	$5,031.00	42	45339.6	289788	7315	252.4	6	11	5
8111	Automotive Repair and maintenance	$9,095.00	45	66582.6	132964	3732	280.7	8	9	5
5311	Lessors of real estate	$8,764.00	46	50760.4	78429	1888	240.7	12	12	7
6111	Elementary and secondary schools	$9,596.00	37	20605.4	875087	16169	184.8	2	22	7
7211	Traveler accommodation	$5,792.00	38	43325.8	162269	3497	215.5	9	17	9
7221	Full-Service restaurants	$4,371.00	24	24673	499789	7879	157.6	4	32	10
5629	Remediation and other waste management services	$14,560.00	72	89842.2	95755	1764	184.2	13	23	10
7139	Other amusement and recreation industries	$6,329.00	33	26585.7	120372	2068	171.8	11	26	12
8113	Commercial and industrial machinery and equipment (except automotive and electronic) repair and maintenance	$8,447.00	43	74734.6	27780	876	315.3	31	7	13
7223	Special food services	$6,427.00	37	47563.6	56334	1223	217.1	24	16	14
7112	Spectator sports	$8,258.00	63	77654.1	13226	649	490.7	41	2	15
**5622**	**Waste treatment and disposal**	**$6,804.00**	**31**	**65461.2**	**13378**	**484**	**361.8**	**45**	**5**	**18**
**7222**	**Limited-Service eating places**	**$4,578.00**	**27**	**15293.1**	**722327**	**7681**	**106.3**	**5**	**49**	**20**

There were 87 NAICS Industry Groups ranked in the PI for All Injury Types.

FTE = (hours/2000); Severity TL = (TL days/10,000 FTE).

Included are industry groups in the Top 5 by Count or Rate Rank (bold) but not in the Top 15 by Prevention Index.

Waste Collection (NAICS 5621) had the highest compensable claim rate per 10,000 FTE for All Injury Types (580.9; Table 2) and the highest severity rate (TL days/10,000 FTE). For SF compensable claims, Remediation and Other Waste Management Services (NAICS 5629) had the highest median cost per compensable claim, $14,560 and highest median time loss (Table 2).

### Work-Related Musculoskeletal Disorder (WMSD)

WMSDs (Table 3) account for approximately 41% of compensable claims in the Services Sector, and had the highest compensable claim rates. For WMSD compensable claims, there were 79 industry groups that met the inclusion criteria. Executive, Legislative, and Other General Government Support (NAICS 9211) was ranked the highest for WMSD compensable claims. Waste Collection (NAICS 5621) had the highest compensable claim rate per 10,000 FTE. In SF compensable claims, the highest median compensable claim cost, median days TL, and severity rate were all found in Remediation and Other Waste Management Services (NAICS 5629) (data not shown). Both Waste Collection and Remediation fall in the 3-digit NAICS subsector 562 - Waste Management and Remediation Services. Subsector 811 - Repair and Maintenance, also has several industry groups, including Automotive Repair and Maintenance, ranked in the Top 15 for WMSD.

**Table 3:** **Top 15 industry groups by prevention index for Work-Related Musculoskeletal Disorders (WMSD) within the services sector in WA (SF + SI), 2002 - 2010**

**4-digit NAICS (industry group) & industry group description**		**FTE**	**# Compensable claims (COUNT)**	**Compensable claim rate/10,000 FTE (RATE)**	**Count rank**	**Rate rank**	**PI rank**
9211	Executive, legislative, and other general government support	655,654	10,247	156.3	1	4	1
5617	Services to buildings and dwellings	215,906	3,393	157.2	3	3	2
5621	Waste collection	38,768	1,031	265.9	10	1	3
9221	Justice, public order, and safety activities	137,032	1,735	126.6	7	8	4
5613	Employment services	289,788	3,003	103.6	4	13	5
8111	Automotive repair and maintenance	132,964	1,566	117.8	9	10	6
5311	Lessors of real estate	78,429	846	107.9	11	12	7
7211	Traveler accommodation	162,269	1,574	97.0	8	15	7
6111	Elementary and secondary schools	875,087	6,345	72.5	2	26	9
5629	Remediation and other waste management services	95,755	845	88.2	12	18	10
8121	Personal care services	76,800	690	89.8	16	17	11
7223	Special food services	56,334	522	92.7	23	16	12
8123	Drycleaning and laundry services	32,347	364	112.5	31	11	13
8113	Commercial and industrial machinery and equipment (except automotive and electronic) repair and maintenance	27,780	345	124.2	33	9	13
8114	Personal and household goods repair and maintenance	20,813	301	144.6	38	5	15
**7112**	**Spectator sports**	**13,226**	**215**	**162.6**	**44**	**2**	**17**
**7222**	**Limited-service eating places**	**722,327**	**2,897**	**40.1**	**5**	**52**	**23**

There were 79 NAICS Industry Groups ranked in the PI for Work-Related Musculoskeletal Disorders.

FTE = (hours/2000).

Included are industry groups in the Top 5 by Count or Rate Rank (bold) but not in the Top 15 by Prevention Index.

### Fall to lower level

For Fall to Lower Level compensable claims (Table 4), there were 39 industry groups that met the inclusion criteria. The median costs, median days TL, and severity rates were higher in Fall to Lower Level than in the other 6 injury types (data not shown), particularly in Personal and Household Goods Repair and Maintenance (NAICS 8114), which includes workers engaged in repair and maintenance of home and garden equipment, appliances, upholstery and furniture, and footwear and leather goods. Both NAICS 811 - Repair and Maintenance and NAICS 531 - Real Estate, which includes renting/leasing real estate and property management and appraisal services, had multiple industry groups represented in Fall to Lower Level.

**Table 4:** **Top 15 industry groups by prevention index for fall to lower level within the services sector in WA (SF + SI), 2002 - 2010**

**4-digit NAICS (industry group) & industry group description**		**FTE**	**# Compensable claims (COUNT)**	**Compensable claim Rate/10,000 FTE (RATE)**	**Count rank**	**Rate rank**	**PI rank**
5617	Services to buildings and dwellings	215,906	918	42.5	2	2	1
9211	Executive, legislative, and other general government support	655,654	1,169	17.8	1	10	2
8111	Automotive repair and maintenance	132,964	256	19.3	7	8	3
5311	Lessors of real estate	78,429	186	23.7	11	5	4
5613	Employment services	289,788	464	16.0	4	14	5
5313	Activities related to real estate	110,988	200	18.0	10	9	6
9221	Justice, public order, and safety activities	137,032	236	17.2	8	12	7
5621	Waste collection	38,768	126	32.5	18	4	8
6111	Elementary and secondary schools	875,087	877	10.0	3	20	9
7211	Traveler accommodation	162,269	227	14.0	9	16	10
7139	Other amusement and recreation industries	120,372	171	14.2	12	15	11
8114	Personal and household goods repair and maintenance	20,813	75	36.0	25	3	12
7112	Spectator sports	13,226	69	52.2	28	1	13
5616	Investigation and security services	83,931	135	16.1	16	13	13
5324	Commercial and industrial machinery and equipment rental and leasing	36,810	79	21.5	24	7	15
**7222**	**Limited-service eating places**	**722,327**	**355**	**4.9**	**5**	**37**	**19**

There were 39 NAICS Industry Groups ranked in the PI for Fall to Lower Level.

FTE = (hours/2000).

Included are industry groups in the Top 5 by Count or Rate Rank (bold) but not in the Top 15 by Prevention Index.

### Fall on Same Level

For Fall on Same Level (Table 5) compensable claims, there were 50 industry groups that met the inclusion criteria. Services to Buildings and Dwellings (NAICS 5617) was ranked highest overall by PI and for claim rate. NAICS Subsector 722 - Food Services and Drinking Places and NAICS 561 - Administrative and Support Services (which includes Employment Services and Investigative and Security Services) are the subsectors with the most representation in the Top 15 for Fall on Same Level.

**Table 5:** **Top 15 industry groups by prevention index for fall on same level within the services sector in WA, 2002 - 2010**

**4-digit NAICS (industry group) & industry group description**		**FTE**	**# Compensable claims (COUNT)**	**Compensable claim rate/10,000 FTE (RATE)**	**Count rank**	**Rate rank**	**PI rank**
5617	Services to buildings and dwellings	215,906	1,006	46.6	5	1	1
7211	Traveler accommodation	162,269	617	38.0	6	4	2
9211	Executive, legislative, and other general government support	655,654	2,180	33.2	2	8	2
6111	Elementary and secondary schools	875,087	2,629	30.0	1	10	4
9221	Justice, public order, and safety activities	137,032	536	39.1	8	3	4
7221	Full-service restaurants	499,789	1,526	30.5	3	9	6
7139	Other amusement and recreation industries	120,372	361	30.0	9	11	7
7223	Special food services	56,334	193	34.3	18	6	8
5621	Waste collection	38,768	160	41.3	23	2	9
8111	Automotive repair and maintenance	132,964	324	24.4	10	16	10
5613	Employment services	289,788	597	20.6	7	20	11
5616	Investigation and security services	83,931	206	24.5	15	15	12
5313	Activities related to real estate	110,988	235	21.2	12	18	12
5311	Lessors of real estate	78,429	204	26.0	16	14	12
7222	Limited-service eating places	722,327	1,329	18.4	4	26	12
**5321**	**Automotive equipment rental and leasing**	**25,744**	**93**	**36.1**	**34**	**5**	**17**

There were 50 NAICS Industry Groups ranked in the PI for Fall on Same Level. SF Data not shown for SF industry groups that do not meet the inclusion criteria alone (without the addition of SI data).

FTE = (hours/2000).

Included are industry groups in the Top 5 by Count or Rate Rank (bold) but not in the Top 15 by Prevention Index.

### Struck By/Against

For Struck By/Against (Table 6) compensable claims, there were 48 industry groups that met the inclusion criteria. Employment Services (NAICS 5613) was ranked the highest for Struck By/Against compensable claims, followed by Services to Buildings and Dwellings (NAICS 5617); these groups are both in the NAICS 561 - Administrative and Support Services subsector. NAICS Subsector 722 - Food Services and Drinking Places was also represented by 3 industry groups in the Top 15.

**Table 6:** **Top 15 industry groups by prevention index for struck by/against within the services sector in WA, 2002 - 2010**

**4-digit NAICS (industry group) & industry group description**		**FTE**	**# Compensable claims (COUNT)**	**Compensable claim rate/10,000 FTE (RATE)**	**Count rank**	**Rate rank**	**PI rank**
5613	Employment services	289,788	1,515	52.3	4	5	1
5617	Services to buildings and dwellings	215,906	1,363	63.1	5	4	1
9211	Executive, legislative, and other general government support	655,654	2,222	33.9	1	12	3
5621	Waste collection	38,768	274	70.7	11	2	3
8111	Automotive repair and maintenance	132,964	651	49.0	7	8	5
7221	Full-service restaurants	499,789	1,537	30.8	3	14	6
8113	Commercial and industrial machinery and equipment (except automotive and electronic) repair and maintenance	27,780	185	66.6	16	3	7
5311	Lessors of real estate	78,429	269	34.3	13	10	8
9221	Justice, public order, and safety activities	137,032	382	27.9	9	16	9
7211	Traveler accommodation	162,269	430	26.5	8	17	9
7112	Spectator sports	13,226	134	101.3	26	1	11
6111	Elementary and secondary schools	875,087	1,681	19.2	2	25	11
7139	Other amusement and recreation industries	120,372	305	25.3	10	19	13
7223	Special food services	56,334	184	32.7	17	13	14
7224	Drinking places (alcoholic beverages)	67,862	199	29.3	15	15	14

There were 48 NAICS Industry Groups ranked in the PI for Struck By/Against.

FTE = (hours/2000).

Fall on Same Level and Struck By/Against are widely distributed, and were the most common occupational injury types following WMSD, making up approximately 13% of compensable claims respectively. These injury types (Tables 5 and 6) also had the 2^nd^ and 3^rd^ highest numbers of industry groups that met the inclusion criteria (groups that report these injury types regularly).

### Caught in/under/between

For Caught In/Under/Between (Table 7) compensable claims, there were 13 industry groups that met the inclusion criteria. Rates and counts for these claims were the lowest among the 7 identified injury types. Employment Services (NAICS 5613) was ranked the highest for Caught/In/Under Between compensable claims overall and by count. Waste Collection (NAICS 5621) ranked the highest by compensable claim rate.

**Table 7:** **Industry groups by prevention index for caught in/under/between within the services sector in WA, 2002 - 2010**

**4-digit NAICS (industry group) & industry group description**		**FTE**	**# Compensable claims (COUNT)**	**Compensable claim rate/10,000 FTE (RATE)**	**Count rank**	**Rate rank**	**PI rank**
5613	Employment services	289,788	422	14.6	1	2	1
5617	Services to buildings and dwellings	215,906	243	11.3	3	3	2
9211	Executive, legislative, and other general government support	655,654	347	5.3	2	6	3
5621	Waste collection	38,768	71	18.3	8	1	4
8111	Automotive repair and maintenance	132,964	135	10.2	5	4	4
6111	Elementary and secondary schools	875,087	226	2.6	4	10	6
5111	Newspaper, periodical, book, and directory publishers	82,937	64	7.7	10	5	7
9221	Justice, public order, and safety activities	137,032	68	5.0	9	7	8
7222	Limited-service eating places	722,327	134	1.9	6	12	9
7221	Full-service restaurants	499,789	115	2.3	7	11	9
7139	Other amusement and recreation industries	120,372	55	4.6	11	8	11
7211	Traveler accommodation	162,269	52	3.2	12	9	12
5413	Architectural, engineering, and related services	262,812	46	1.8	13	13	13

There were 13 NAICS Industry Groups ranked in the PI for Caught In/Under/Between. SF Data not shown for SF industry groups that do not meet the inclusion criteria alone (without the addition of SI data).

FTE = (hours/2000).

### Motor vehicle

For Motor Vehicle (Table 8) compensable claims, there were 20 industry groups that met the inclusion criteria. Executive, Legislative, and Other General Government Support (NAICS 9211) was ranked the highest for Motor Vehicle compensable claims. Automotive Equipment Rental and Leasing (NAICS 5321) had the highest compensable claim rate.

**Table 8:** **Top 15 industry groups by prevention index for motor vehicles within the services sector in WA SF, 2002 - 2010**

**4-digit NAICS (industry group) & industry group description**		**FTE**	**# Compensable claims (COUNT)**	**Compensable claim rate/10,000 FTE (RATE)**	**Count rank**	**Rate rank**	**PI rank**
9211	Executive, legislative, and other general government support	655,654	1,367	20.8	1	3	1
5617	Services to buildings and dwellings	215,906	417	19.3	3	4	2
5621	Waste collection	38,768	96	24.8	8	2	3
9221	Justice, public order, and safety activities	137,032	172	12.6	4	6	3
5321	Automotive equipment rental and leasing	25,744	75	29.1	11	1	5
5616	Investigation and security services	83,931	98	11.7	6	7	6
6111	Elementary and secondary schools	875,087	436	5.0	2	12	7
8111	Automotive repair and maintenance	132,964	93	7.0	9	9	8
9261	Administration of economic program	50,494	67	13.3	13	5	8
5614	Business support services	168,586	78	4.6	10	13	10
7222	Limited-service eating places	722,327	166	2.3	5	18	10
5613	Employment services	289,788	97	3.3	7	16	10
9241	Administration of environmental quality programs	50,862	48	9.4	17	8	13
8129	Other personal services	90,669	51	5.6	16	10	14
5111	Newspaper, periodical, book, and directory publishers	82,937	46	5.5	18	11	15

There were 20 NAICS Industry Groups ranked in the PI for Motor Vehicle. SF Data not shown for SF industry groups that do not meet the inclusion criteria alone (without the addition of SI data).

FTE = (hours/2000).

### Overexertion

For Overexertion (Table 9) compensable claims, there were 24 industry groups that met the inclusion criteria. Services to Buildings and Dwellings (NAICS 5617) ranked the highest overall. Waste Collection (NAICS 5621) had the highest compensable claim rate. The NAICS 811 subsector - Repair and Maintenance - was highly represented in the Top 15 for Overexertion.

**Table 9:** **Top 15 industry groups by prevention index for overexertion within the services sector in WA, 2002 - 2010**

**4-digit NAICS (industry group) & industry group description**		**FTE**	**# Compensable claims (COUNT)**	**Compensable claim rate/10,000 FTE (RATE)**	**Count rank**	**Rate rank**	**PI rank**
5617	Services to buildings and dwellings	215,906	357	16.5	3	4	1
9211	Executive, legislative, and other general government support	655,654	807	12.3	1	7	2
5621	Waste collection	38,768	111	28.6	9	1	3
5613	Employment services	289,788	353	12.2	4	8	4
8111	Automotive repair and maintenance	132,964	213	16.0	7	5	4
6111	Elementary and secondary schools	875,087	574	6.6	2	13	6
5311	Lessors of real estate	78,429	106	13.5	11	6	7
7211	Traveler accommodation	162,269	126	7.8	8	11	8
9221	Justice, public order, and safety activities	137,032	108	7.9	10	10	9
7221	Full-service restaurants	499,789	273	5.5	5	17	10
5313	Activities related to real estate	110,988	82	7.4	12	12	11
8113	Commercial and industrial machinery and equipment (except automotive and electronic) repair and maintenance	27,780	48	17.3	22	3	12
8114	Personal and household goods repair and maintenance	20,813	45	21.6	24	2	13
7223	Special food services	56,334	57	10.1	17	9	13
7222	Limited-service eating places	722,327	221	3.1	6	21	15

There were 24 NAICS Industry Groups ranked in the PI for Overexertion. SF Data not shown for SF industry groups that do not meet the inclusion criteria alone (without the addition of SI data).

FTE = (hours/2000).

Complete tables of industry group rankings for All Injury Types and each injury type, as well as expanded tables with compensable claim costs, time loss, and severity rates (SF compensable claims data only) are available upon request.

## Discussion

Using the PI to rank industry groups within the Services Sector in WA identified industry groups that may most benefit from research and prevention activities. These injury types account for the majority of compensable claims, claims costs, and time loss. Industry groups or subsectors that appear highly ranked by PI across injury types have a higher burden of occupational injury. These injuries can be personally devastating and are associated with high costs, both in direct workers’ compensation claim costs and time loss wage replacement, and in costs to the worker, employer, health-care systems, and society. Several industry groups were consistently ranked highly (Table 10) across injury types, indicating that they warrant priority focus in overall injury prevention, including: Services to Buildings and Dwellings (NAICS 5617), Executive, Legislative, and Other General Government Support (NAICS 9211), Waste Collection (NAICS 5621), Employment Services (NAICS 5613) and Automotive Repair and Maintenance (NAICS 8111).

**Table 10:** **Top 15 services sector industry groups that were highest ranked by prevention index for 7 common injury types, WA, 2002 - 2010**

**4-digit NAICS (industry group) & industry group description**		**Average of PI ranks**
5617	Services to buildings and dwellings*	1.6
9211	Executive, legislative, and other general government support*	2.1
5621	Waste collection*	4.9
5613	Employment services*	5.6
8111	Automotive repair and maintenance*	6.0
9221	Justice, public order, and safety activities*	6.6
6111	Elementary and secondary schools*	7.6
7211	Traveler accommodation	7.7
5311	Lessors of real estate	8.0
9261	Administration of economic program	9.0
5616	Investigation and security services	10.7
7223	Special food services	12.0
7221	Full service restaurants*	12.3
8113	Commercial and industrial machinery and equipment (except automotive and electronic) repair and maintenance	12.3
5313	Activities related to real estate	13.2
**7139**	Other amusement and recreation industries*	**14.3**
**7222**	Limited service eating places*	**15.9**

*Indicates that industry group was ranked in the Top 25 overall and for all seven injury types.

Included are industry groups (bold) not in the Top 15, but ranked in the Top 25 overall and for all seven injury types.

Services to Buildings and Dwellings (NAICS 5617) was ranked in the top 2 in all 7 injury types (Tables 3, 4, 5, 6, 7, 8 and 9) and ranked highest by average of PI ranks (Table 10). Workers in this industry group are at high risk for occupational injury across the board and would benefit substantially from injury prevention and safety improvements. The Services to Buildings and Dwellings industry group includes: Exterminating and Pest Control Services, Janitorial Services, Landscaping Services, Carpet and Upholstery Cleaning Services, and Other Services to Buildings and Dwellings (e.g. chimney cleaning, drain cleaning). These workers may encounter multiple hazards, work alone, or work with hazardous tools or chemicals. They may also face low wages, and other job stresses in addition to risks for a variety of occupational injuries and illnesses [16]. Workers in Services to Buildings and Dwellings also reported the greatest number of occupational traumatic injury fatalities of industry groups within the NORA Services Sector during 2003–2007 [5]. Services to Buildings and Dwellings has been previously identified in WA [2,12] as a high-risk industry group, and continued high rankings across injury types establish it as a group in need of prevention and intervention efforts.

Employment Services (NAICS 5613) includes establishments that provide staff or refer and/or place applicants, where work activities and control over workplace hazards is generally the responsibility of the hiring employer, but the workers compensation liability rests with the temporary agency. Temporary Help workers have been previously identified by WA as a group in need of research and prevention efforts, due to increased injury rates [17-19]. In the same NAICS Subsector (561), Investigative and Security Services (NAICS 5616), was also highly ranked by average of PI scores across injury types (Table 10) and was at high risk for Fall injuries and for Motor Vehicle incidents.

Two industry groups from NAICS 562 - Waste Management and Remediation Services, a subsector engaged in local hauling of waste materials, operating materials recovery facilities (including sorting), and providing remediation (cleaning contaminated buildings, mines, soil, water) and septic services, were identified as being especially high-risk for common and costly injuries. Waste Collection (NAICS 5621) had the highest rate and severity rate for All Injury Types and was consistently ranked highly (Table 10), especially by rate, for prevention efforts in each of the identified injury types. Waste Collection has been identified previously and is already targeted for research and prevention efforts in WA [12,20], which identified WMSDs as a primary injury type for the waste management industry and ‘Containers’ as the most common source of injury [20]. Both Waste Collection and Remediation and Other Waste Management Services, along with another in this subsector, Waste Treatment and Disposal (NAICS 5622), have also been identified in the top 5 Services Sector Industry Groups by average annual DAFW rates [5].

Automotive Repair and Maintenance (NAICS 8111), which includes body shops and collision repair work, was ranked highly across all 7 injury types and was ranked 5^th^ overall by average PI rank (Table 10). Collision repair shops, in addition to ranking highly for these seven occupational injury types, have been previously identified in WA for occupational respiratory illness (isocyanates asthma) prevention activities [21].

Additional industry groups may be targeted by specific injury type(s), such as Fall to Lower Level, which remains a leading cause of injury. A study of occupational ladder fall injuries (using the Bureau of Labor Statistics Survey of Occupational Injuries and Illnesses (SOII) data) reported that 29% of workers with occupational ladder fall injuries were in a Services industry [22]. Fall on Same Level injuries are also widespread in the Services sector. Accommodation and Food Services workers (NAICS 72, included in the NORA Services Sector) experienced the highest number of falls over a five-year period and had higher Fall on Same Level rates than the construction industry based on United States Bureau of Labor Statistics data [23]. Fall to Lower Level and Fall on Same Level together accounted for approximately 20% of compensable claims in the WA Services Sector between 2002–2010.

Subsectors NAICS 562 - Waste Management and Remediation and NAICS 811 - Repair and Maintenance, have several industry groups in the Top 15 for WMSDs (Table 3), and Repair and Maintenance is also highly represented in the Top 15 for Overexertion (Table 9). WMSDs and Overexertion can be caused or aggravated by work exposures and activities such as material handling, awkward postures, and repetitive or forceful exertions; prevention efforts in industry groups in these subsectors should be directed towards reducing such exposures.

Another factor in the health of Services Sector workers is that many of these jobs are low or minimum wage work. Data from the Bureau of Labor Statistics (BLS) in 2012 show that the industry with the highest proportion of hourly paid workers at or below the federal minimum wage was “Leisure and Hospitality” [24] which would include workers in NAICS 72 - Accommodation and Food Services and 71 - Arts, Entertainment, and Recreation. Five industry groups that are high-ranking across injury types by average PI (Table 10) are found in NAICS 71–72. A study of low socio-economic status (SES) workers found that they reported more chronic diseases, increased risk behaviors, and worse health; were disproportionately younger, single, non-white and Spanish speaking; and clustered in low-wage industries, two of which are Services Sector industries - Accommodation and Food Services and Administrative and Waste Services [25]. In the same study, half of Services Sector industries had median annual wages below $35,000 in 2008; including: Accommodation and Food Services; Arts, Entertainment, and Recreation; Administrative and Waste Services; Other Services; and Real Estate and Rental and Leasing [25]. Using BLS Occupational Employment Wage Estimates from May 2010, for median hourly wage and mean annual wage for the U.S. (national, all occupations) [26] compared with those industries in the Service Sector [27], this pattern remained. Industry groups from these sectors - Accommodation and Food Services, Arts, Entertainment, and Recreation, Administrative and Waste Services, Other Services, and Real Estate and Rental and Leasing - made up the majority of industry groups that were highly ranked for prevention across injury types (Table 10); only 4 industry groups (3 from NAICS 92 - Public Administration, and one from NAICS 61 - Educational Services) were from industry sectors with higher median hourly/mean annual wages. Accommodation and Food Services includes workers in Traveler Accommodation (NAICS 7221) which was also ranked highly by PI across several injury types (Table 10). Traveler Accommodation includes Hotels and Motels, Casino Hotels, and Other Traveler Accommodations (bed and breakfast inns, guest houses, cabins and cottages, tourist homes, hostels). Hotel workers in particular, have been identified as having higher injury rates, especially for females, housekeepers, and those of Hispanic ethnicity [28]. These jobs have also been found to be associated with cleaning tasks and high physical effort (strain, muscle load, postures, repetitive movement), low wages and low job control, among other risks [28]. Workers in Services to Buildings and Dwellings who may face similar risks include Janitors and Cleaners and Landscaping workers.

While many industries in the Services Sector have much higher median hourly and mean annual wages, these industries are not as strongly represented in the PI rankings for work-related injuries in WA. Low socioeconomic status is also associated with job insecurity and work organization hazards [29]. In contrast, those ranked lowest by PI (data not shown) for All Injury Types were clustered in the Finance and Insurance, Information, and Professional, Scientific, and Technical Services industries, which were among the industries with the highest reported wages in the Services Sector, according to the 2010 BLS data [27].

There are relatively few studies in the literature that rank industries by some measure of risk, count or cost of occupational injury and illness. A previous study [3] ranking industries (using Standard Industrial Classification System (SIC) codes [30]) for estimated high-cost fatal and non-fatal injuries and illnesses in the United States identified many industries similar to the findings in the WA Services Sector: including Services to Buildings, Personnel Supply Services, and Automotive Repair Shops. Okun et al. [4] used a ‘risk index score’ and also identified (by SIC code) landscape and horticultural services (SIC 078), which may be comparable to some workers in NAICS 5617 Services to Buildings and Dwellings (e.g. NAICS 561730 Landscaping Services). However, the study’s difference in classification system and the restriction to ‘small-business’ makes comparison difficult.

A recent study characterizing occupational injuries, illnesses and fatalities in Services Sector workers [5] using BLS data had results that were similar to the WA data. Industry groups were ranked by average annual rank of Days-Away-From-Work (DAFW) Injury Rates (for several years), and identified similar industry groups. The five highest ranked industry groups by DAFW were NAICS 5621 Waste Collection, 5622 Waste Treatment and Disposal, 5323 General Rental Centers, 5617 Services to Buildings and Dwellings, and 5629 Remediation and Other Waste Management [5]. The same study [5] also used a PI to rank Services Sector industry groups combining DAFW Injury and Illness Counts and Rates, and identified 5617 Services to Buildings and Dwellings and 5621 Waste Collection, ranked 1^st^ and 3^rd,^ respectively.

The PI results represent a portion of the data, and the difference between individual ranks does not reflect the magnitude of the actual difference in count or rate between industry groups. The difference between individual ranks is of less importance than which industry groups are consistently ranked highly (demonstrating higher burden of injury).

The particular combination of compensable claim rate and rank may suggest different prevention activities for the industry or industries at risk and facilitate decision making as to the approach for allocation of prevention resources. For example, in the WMSD PI (Table 3), Services to Buildings and Dwellings (NAICS 5617) has a high rate rank and high count rank, which indicates that it is a large, hazardous industry group that could benefit from an intensive multi-faceted approach using every available resource (consultation, enforcement, and education). In the same table (Table 3), Elementary and Secondary Schools (NAICS 6111) has the largest number of FTEs and is ranked 2^nd^ for compensable claims but has one of the lower compensable claims rates of all the industry groups that met the inclusion criteria for this injury type. High count rank but low rate rank may indicate a large, but less hazardous, industry, with claims scattered across a large number of workplaces and it is unlikely that any single workplace has high risk; therefore, the industry group may be more suited to an education campaign in order to reach the large number of workplaces and employees. Conversely, smaller industry groups with low count but high rate, such as Waste Treatment and Disposal (NAICS 5622, Table 3) may be hazardous industries with concentrated risk; a focused inspection/consultation may be an effective approach. Industry groups with low claim count and low claim rate likely need minimal prevention resources or efforts, with injury and illness surveillance for any emerging hazards. Business size may also influence strategies for prevention, as employer size varies considerably in the Services Sector. Large organizations may have more resources to devote to occupational injury prevention, while small businesses may benefit from collaboration with intermediary organizations to help develop and spread health and safety interventions [31].

Several of the limitations associated with this PI methodology and the use of WC data have been previously discussed [2]. Generalizability of results is limited by the varying statutes of workers’ compensation in the United States, as well as by variations in industry distribution. Another limitation to this report is that the injury and illness rates reported in this study are dependent on the completeness of reporting of cases and employee work hours to the WC system. These may be incomplete for a variety of reasons (e.g. employer reporting practices, fear of retribution, administrative barriers, or the use of alternative medical insurance providers). The extent of underreporting to the WA WC system is unknown. Additionally, data coding in large administrative databases such as the WA WC system is not always complete or accurate and there is a chance for miscoding (for example, OIICS coding takes place at the initial assessment of the claim, and the injury or illness may be poorly defined on the initial claim form, and thus the coding may not reflect the true nature of the injury or illness associated with the claim). Future efforts to rank industry groups for prevention and intervention activities may include other elements (an expanded PI approach) such as severity measures based on TL and cost data, to better capture the magnitude of the burden of injury.

PI rankings provide evidence as to where research and prevention efforts can be most effectively targeted for maximum benefit in reducing common and costly injuries. Ranking industries by PI overall and by specific injury types provides data to prioritize industry groups, guide prevention efforts, and inform the setting of policy and research agendas that reflect the particular needs of workers in industry groups within the Services Sector.
